# Prenatal Diagnosis of Compound Heterozygous Beta-Thalassemia: A Report of Two Cases

**DOI:** 10.7759/cureus.107581

**Published:** 2026-04-23

**Authors:** Sangam Jha, Sharda Jha, Vikas C Jha, Aditi Narayan, Raveena Bhagat

**Affiliations:** 1 Obstetrics and Gynecology, All India Institute of Medical Sciences, Patna, IND; 2 Neurosurgery, All India Institute of Medical Sciences, Patna, IND

**Keywords:** amniocentesis, compound heterozygosity, hbb c.126_129del (β⁰), hbb c.79g>a (hbe), hbb c.92+5g>c (β⁰), hbb c.92g>c (β⁺), β-thalassemia

## Abstract

Beta (β)-thalassemia and related hemoglobinopathies remain a major genetic health burden in India. Prenatal diagnosis plays a crucial role in preventing recurrence of severe disease in high-risk couples, particularly those with previously affected children. We report two cases of prenatal diagnosis performed by mid-trimester amniocentesis in couples with prior children affected by severe β-globin disorders. In Case 1, molecular analysis of fetal DNA revealed compound heterozygosity for HBB c.92G>C (β⁺) and HBB c.126_129del (β⁰), consistent with severe β-thalassemia. In Case 2, fetal testing demonstrated HbE/β-thalassemia due to compound heterozygosity for HBB c.79G>A (HbE) and HBB c.92+5G>C (β⁰). Both diagnoses were established using PCR-based amplification and Sanger sequencing after exclusion of maternal DNA contamination. Given the severe clinical phenotype observed in previously affected siblings and following detailed genetic counseling, both couples opted for medical termination of pregnancy. These cases underscore the importance of molecular prenatal diagnosis in accurately identifying severe compound heterozygous hemoglobinopathies. Early carrier detection, definitive fetal genotyping, and timely counseling remain essential strategies for reducing the burden of β-thalassemia in high-prevalence regions.

## Introduction

Thalassemia is an inherited hemoglobin disorder caused by an imbalance in the synthesis of alpha (α)- and beta (β)-globin chains, leading to reduced red blood cell survival and chronic hemolytic anemia [1]. Worldwide, β-thalassemia is one of the most common inherited blood disorders, with the highest prevalence in the Middle East, the Mediterranean region, Central and South Asia, and Africa [2]. Carrier prevalence ranges from 10% to 20% in sub-Saharan Africa, approximately 40% in the Middle East and India, and up to 80% in northern Papua New Guinea and parts of North-East India [3].

Beta-thalassemia results from mutations affecting the β-globin gene, leading to reduced or absent synthesis of β-globin chains. More than 200 mutations have been identified globally, of which approximately 20-21 account for the majority of cases. These include point mutations, insertions, and deletions [4]. The mutations are classified as β⁺, which reduces β-globin synthesis, or β⁰, which completely stops β-globin production.

Based on clinical severity, β-thalassemia is classified into β-thalassemia minor (carrier state), β-thalassemia intermedia, and β-thalassemia major. Patients with β-thalassemia major require lifelong regular blood transfusions and iron chelation therapy [5]. Disease severity depends on the type and combination of inherited mutations, with genotypes such as β⁺β⁺, β⁺β⁰, and β⁰β⁰, producing varying clinical phenotypes.

Prenatal diagnosis plays an important role in preventing the birth of affected children by enabling at-risk couples to make informed reproductive decisions. Advances in diagnostic techniques, such as chorionic villus sampling (CVS), amniocentesis, and molecular genetic testing, have significantly improved early detection and genetic counseling.

This case series describes two cases that highlight real-world experiences with prenatal diagnostic practices and emphasize the importance of early detection, molecular diagnosis, and timely prenatal testing in families with compound heterozygous β-thalassemia, thereby supporting informed reproductive decision-making.

## Case presentation

Case 1

A 32-year-old G2P1L1 woman presented at 14 weeks of gestation with concerns about the recurrence of β-thalassemia, as both parents were known carriers. Their first child, now four years old, was healthy at birth but developed progressive pallor, poor feeding, and hepatosplenomegaly by six months of age. The child was diagnosed with β-thalassemia major. Molecular testing revealed compound heterozygosity for HBB c.92G>C (exon 1) and HBB c.126_129del, with each parent carrying one mutation.

The child was started on regular blood transfusions and iron chelation therapy, along with folic acid supplementation and long-term hematology follow-up. The parents received counseling regarding disease management, prognosis, and the importance of prenatal testing in future pregnancies.

During the current pregnancy, the mother had no significant medical issues. After genetic counseling, the couple was informed of a 25% recurrence risk. Due to financial constraints and the definitive nature of testing, amniocentesis was performed at 15 weeks of gestation. Informed consent was obtained, and fetal cardiac activity was confirmed by ultrasound prior to the procedure. Amniocentesis was performed under aseptic conditions with a single needle insertion, and 20 mL of amniotic fluid was collected for genetic testing. Extracted DNA from maternal blood and fetal samples underwent quality assessment, and fetal DNA was confirmed to be free from maternal contamination. Mutation analysis was performed using polymerase chain reaction (PCR) and Sanger sequencing. The fetal sample demonstrated compound heterozygosity for HBB c.92G>C (β⁺) and HBB c.126_129del (β⁰). This β⁺β⁰ genotype is associated with severe β-thalassemia, clinically similar to β-thalassemia major. The c.92G>C mutation results in reduced β-globin production due to abnormal splicing, while the c.126_129del mutation, a four-base pair deletion, causes a frameshift and complete loss of β-globin synthesis from the affected allele. Overall, β-globin production is therefore markedly reduced. Following counseling, medical termination of pregnancy was undertaken.

Case 2

A 28-year-old G3P1L1A1 woman presented at 12 weeks of gestation with a history of a child with thalassemia major. Both parents were known carriers based on high-performance liquid chromatography (HPLC) findings.

Their seven-year-old child had a history of easy fatigability, progressive pallor, and abdominal distension over the past year. The child had been apparently well until approximately 18 months of age, when pallor and mild jaundice were first noted. Due to limited access to healthcare, only intermittent iron supplementation was received. Over time, the child’s exercise tolerance declined, with reduced ability to participate in play. Antenatal screening had not been performed during that pregnancy, and delivery was at term via an uncomplicated vaginal birth. Developmental milestones were achieved appropriately.

Laboratory investigations, including hemoglobin analysis by HPLC, showed HbF 34%, HbE + HbA2 56.2%, and HbA 5.8%. This pattern suggested a compound heterozygous state for HbE and β-thalassemia rather than HbE trait alone. Molecular analysis of the β-globin (HBB) gene confirmed heterozygosity for HBB c.79G>A (Glu26Lys), consistent with HbE, and a β⁰-thalassemia mutation (HBB c.92+5G>C), establishing the diagnosis of HbE/β-thalassemia. Based on clinical and laboratory findings, the child was classified as having a moderately severe form of HbE/β-thalassemia and required intermittent blood transfusions.

The parents presented for prenatal diagnosis in the current pregnancy and received genetic counseling. Hemoglobin analysis showed that the mother had HbE trait (HbA 68%, HbE + HbA2 28%, HbF 4%), while the father had β-thalassemia trait (HbA 92%, HbA2 5.2%, HbF 2.8%). Amniocentesis was performed at 16 weeks of gestation. Informed consent was obtained, and fetal cardiac activity was confirmed by ultrasound prior to the procedure. Under aseptic conditions, 20 mL of amniotic fluid was collected and sent for genetic analysis. DNA extracted from maternal and fetal samples underwent quality assessment, and fetal DNA was confirmed to be free from maternal contamination. Target-specific PCR amplification followed by Sanger sequencing identified compound heterozygosity for HBB c.92+5G>C and HBB c.79G>A (Glu26Lys). This genotype is consistent with HbE/β-thalassemia, similar to that observed in their previous child. After counseling, the parents opted for medical termination of pregnancy due to concerns about managing another affected child.

## Discussion

Beta-thalassemia and related hemoglobinopathies constitute a significant genetic health burden in India, with carrier rates varying across regions and communities. National data indicate an overall carrier prevalence of approximately 3%-4%, with certain populations exhibiting even higher frequencies; HbE trait is especially prevalent in northeastern and eastern regions, while β-thalassemia trait is widespread across multiple states [3]. The severity of thalassemia correlates with the number and type of globin gene mutations present. Unlike α-thalassemia, where severity depends mainly on the presence of gene deletions, β-thalassemia shows wide variability due to mutations that reduce or inhibit protein synthesis to various extents [6]. Identified HBB mutations and their functional impact are summarized in Table 1.

**Table 1:** Description of identified HBB mutations and their functional impact Source: [6].

Mutation	Location	Mutation type	Functional Effect	Classification
HBB c.79G>A (p.Glu26Lys)	Exon 1	Missense + abnormal splicing	Produces HbE with reduced β-globin synthesis	β⁺
HBB c.92G>C	Exon 1	Splice-affecting point mutation	Reduced β-globin production (10–30%)	β⁺
HBB c.92+5G>C	Intron 1	Splice-site mutation	Abnormal mRNA splicing with residual β-globin	β⁺/β⁰ (functionally severe)
HBB c.126_129del	Exon 2	Frameshift deletion	Premature stop codon, no β-globin production	β⁰

Compound heterozygosity occurs when two different defective alleles are inherited, resulting in an intermediate or severe phenotype, depending on the type of mutation. Studies from Indian prenatal diagnosis programs have shown that compound heterozygous genotypes, such as β⁺/β⁰ and HbE/β-thalassemia, form a substantial proportion of affected fetuses diagnosed by invasive testing, with IVS-I-5 (c.92+5G>C) emerging as one of the most common β-thalassemia mutations [7,8], and HbE (c.79G>A, a missense mutation ) frequently encountered in HbE/β-thalassemia cases [9]. When combined with β⁺ or β⁰ alleles, a wide spectrum of disease severity is produced. The HBB c.126_129del mutation is a β⁰ mutation that completely stops the β-globin synthesis due to a frameshift and premature stop codon [10]. When combined with a β⁺ allele, the resulting phenotype often results in thalassemia intermedia or major. Both cases represent compound heterozygous β-globin disorders, namely, β⁺/β⁰ thalassemia and HbE/β-thalassemia (genotype-phenotype correlation in the presented cases is summarized in Table 2). Although β⁺ mutations are often considered milder, their combination with β⁰ mutations results in severely compromised β-globin synthesis, producing a clinical phenotype indistinguishable from β-thalassemia major. This is well illustrated by the affected siblings in Case 1, who required long-term hematological care. These observations align with the reported cases, underlining the genotype-phenotype correlation where compound heterozygosity resulted in severe clinical phenotypes similar to β-thalassemia major [10].

**Table 2:** Genotype-phenotype correlation in the presented cases Source: [9].

Case	Genotype	Mutation type	Beta globin production	Phenotype
Case 1 (fetus)	c.92G>C / c.126_129del	β⁺/β⁰	Severely reduced	β-thalassemia major-like
Previous child	c.92G>C / c.126_129del	β⁺/β⁰	Severely reduced	β-thalassemia major
Case 2 (fetus)	c.79G>A / c.92+5G>C	HbE/β⁰	Moderately reduced	HbE/β-thalassemia
Previous child	c.79G>A / c.92+5G>C	HbE/β⁰	Moderately reduced	HbE/β-thalassemia (moderately severe)

Disease severity is further influenced by the presence of genetic modifiers, such as BCL11A, HBS1L-MYB, and KLF1, which can increase fetal hemoglobin levels and thus partially compensate for β-globin deficiency [11].

While HPLC is an excellent screening tool, it cannot reliably predict disease severity or recurrence risk. In Case 2, the elevated HbF and HbE/A₂ fraction suggested HbE/β-thalassemia, but molecular confirmation was essential to establish the diagnosis and enable accurate prenatal counseling. This emphasizes that DNA-based testing remains the gold standard for prenatal diagnosis.

Amniocentesis performed in the mid-trimester proved to be a safe, feasible, and definitive diagnostic modality, particularly in resource-limited settings where non-invasive prenatal testing may not be affordable or informative for single-gene disorders. Strict quality control measures, including exclusion of maternal contamination and use of Sanger sequencing, ensured diagnostic accuracy.

Overall, these observations support the importance of carrier screening, molecular diagnosis, and prenatal testing as effective strategies to reduce the clinical, psychosocial, and economic burden of β-thalassemia in high-prevalence areas.

## Conclusions

This case series highlights the wide clinical presentations and molecular heterogeneity of β-thalassemia resulting from different combinations of HBB gene mutations, including β⁺, β⁰, and HbE alleles. The presented cases demonstrate how compound heterozygosity can lead to disease severity ranging from mild anemia to classical transfusion-dependent β-thalassemia major. Prenatal diagnosis played a pivotal role in both cases by enabling early identification of affected fetuses and supporting informed reproductive decision-making.

## References

[1] Musallam KM, Cappellini MD, Coates TD (2024). Αlpha-thalassemia: a practical overview. Blood Rev.

[2] Rao E, Kumar Chandraker S, Misha Singh M, Kumar R (2024). Global distribution of β-thalassemia mutations: an update. Gene.

[3] Williams TN, Weatherall DJ (2012). World distribution, population genetics, and health burden of the hemoglobinopathies. Cold Spring Harb Perspect Med.

[4] Shafique F, Ali S, Almansouri T (2021). Thalassemia, a human blood disorder. Braz J Biol.

[5] Jagannath VA, Fedorowicz Z, Al Hajeri A, Hu N, Sharma A (2011). Hematopoietic stem cell transplantation for people with ß-thalassaemia major. Cochrane Database Syst Rev.

[6] Danjou F, Anni F, Perseu L (2012). Genetic modifiers of β-thalassemia and clinical severity as assessed by age at first transfusion. Haematologica.

[7] Agarwal S, Tamhankar PM, Kumar R, Dalal A (2010). Clinical and haematological features in a compound heterozygote (HBB:c.92 + 5G &gt; C/HBB:c.93-2A &gt; C) case of thalassaemia major. Int J Lab Hematol.

[8] Bhattacharjee S, Ghosh S, Ray R, Bhattacharyya M (2023). Rare mutations in the beta-globin gene and their clinical phenotypes. J Hematol Allied Sci.

[9] Dolai TK, Dutta S, Bhattacharyya M, Ghosh MK (2012). Prevalence of hemoglobinopathies in rural Bengal, India. Hemoglobin.

[10] Colah R, Nadkarni A, Gorakshakar A (2018). Prenatal diagnosis of HbE-β-thalassemia: experience of a center in Western India. Indian J Hematol Blood Transfus.

[11] Qadah T, Noorwali A, Alzahrani F, Banjar A, Filimban N, Felimban R (2020). Detection of BCL11A and HBS1L-MYB genotypes in sickle cell anemia. Indian J Hematol Blood Transfus.

